# Liquid Hot Water and Steam Explosion Pretreatment Methods for Cellulosic Raw Materials: A Review

**DOI:** 10.3390/polym17131783

**Published:** 2025-06-27

**Authors:** Evgenia K. Gladysheva

**Affiliations:** Bioconversion Laboratory, Institute for Problems of Chemical and Energetic Technologies, Siberian Branch of the Russian Academy of Sciences (IPCET SB RAS), 659322 Biysk, Russia; evg-gladysheva@yandex.ru

**Keywords:** liquid hot water, steam explosion, cellulosic raw materials, pretreatment methods

## Abstract

Cellulosic raw materials are the most common source of carbon on Earth and are in great demand for the production of high-value-added products. Cellulosic feedstocks represent a strong matrix consisting of cellulose, lignin, and hemicelluloses. The efficient transformation of cellulosic raw materials into fermentable sugars requires the use of effective pretreatment strategies. The methods employed for pretreatment should be efficient, have low operating costs, and exhibit lower environmental impact. The present review describes pretreatment methods like liquid hot water (LHW) and steam explosion (SE) and highlights peculiar features, benefits and disadvantages of these processes. The effectiveness of these pretreatment methods and their effect on cellulosic raw materials strongly depends on the type of feedstock (component composition), pretreatment method, and pretreatment conditions (pressure, temperature, time, etc.). The LHW pretreatment requires neither addition of chemicals and catalysts nor grinding stage, but requires high energy inputs. The SE pretreatment is regarded as environmentally friendly and requires lower energy inputs, but contributes to the formation of toxic compounds. The life cycle assessment approach demonstrated that the SE pretreatment outperforms dilute acid pretreatment methods and allows the reduction of energy inputs, thereby improving the environmental performance of the process, while the LHW method improves long-term energy security and creates a greener future.

## 1. Introduction

The use of renewable sources of cellulosic raw materials to produce high-value-added products is the subject matter of global research, due to the growing demand for energy, shortage of fuel reserves, and global climate change. The biorefinery concept is under development in order to produce bio-based products from cellulosic feedstocks to meet the needs of society, including energy security and environmental concerns [1]. Cellulosic raw materials are the most common source of carbon on Earth, but their composition may vary depending on the plant species, harvest season, and geographical location. It is well-known that cellulosic raw materials in their native form represent a matrix consisting of cellulose (38–50%), lignin (10–25%), and hemicelluloses (23–32%) [2].

Cellulose has the basic structure of a linear polymer consisting of D-glucose units linked by β-(1,4)-glycosidic bonds. The hydrogen and van der Waals bonds are the primary links that connect cellulose polymers to form microfibrils, which exist in the crystalline and amorphous forms. The fibrous parts of crystalline cellulose are connected by non-covalent hydrogen bonds and possess lower degradability compared to the amorphous part [1].

Hemicelluloses—as the second most found, heterogeneously branched polymer—are composed of several components, including pentoses (D-xylose and L-arabinose), hexoses (D-glucose, D-mannose, D-galactose), acetyl groups, and uronic acids. The branched structure and the presence of acetyl group govern the lack of crystalline structure of hemicellulose. Moreover, due to its amorphous nature, hemicellulose is easily degradable and, hence, its composition varies depending on the plant species. Acting as a physical barrier, hemicellulose limits the cleavage of cellulose by cellulases [1].

Lignin represents an amorphous polyphenolic polymer with the most recalcitrant structure. Lignin is typically made up of three methoxylated p-hydroxyphenyl propanoid units (monolignols; i.e., p-coumaryl, coniferyl and sinapyl alcohols). The lignin composition, physical distribution and the syringyl (S)-to-guaiacyl (G) unit ratio also have a significant negative impact on cellulase-catalyzed hydrolysis because lignin acts as a physical barrier, limiting the accessibility of cellulose [2,3].

Plant raw materials possess “biomass resistance” which depends on the plant tissue structure, complexity of cell wall components, lignification degree, crystallinity degree, polymerization degree, and biomass heterogeneity [4].

Therefore, in order to utilize cellulosic biomass carbohydrates as much as possible, effective pretreatment strategies should be used, as this is the key to overcoming the “biomass resistance”. The efficiency of transformation of cellulosic feedstocks into fermentable sugars depends on the pretreatment method of the initial feedstock [5]. The purpose of pretreatment is to alter the structural and compositional obstacles, such as porous structure (significant factors are pore size and volume, particle size and specific surface area), chemical composition (relevant factors are hemicelluloses, lignin, acetyl group, and pectin) and cellulose structure (significant factors are cellulose crystallinity and polymerization degree) [6], and to thus improve the hydrolysis rate and increase the yield of fermentable sugars [7]. Cellulose-derived sugars have a significant potential for the production of biofuel (e.g., cellulosic ethanol), chemicals, and biomaterials [8]. In this way, various products can be obtained from cellulosic raw materials through biorefinery.

Various pretreatment types, including physical pretreatment (grinding, microwave), chemical pretreatment (alkaline, acidic and ionic liquids), physicochemical pretreatment (steam explosion, liquid hot water, and ammonia fiber explosion) and biological pretreatment have been developed over the last few decades [8]. Pretreatment should meet the following criteria: minimal need for chemicals; no need for grinding; no formation of inhibitors; increased surface area—extraction and conversion of lignin into byproducts; minimal sugar loss; and minimal reactor cost [9]. Most of the pretreatment methods have drawbacks like complicated process organization, long duration, high investment costs, and high emissions of chemicals [10,11,12]. Therefore, researchers have concentrated much attention on developing more efficient pretreatment methods with lower costs and lower environmental impact [13]. Liquid hot water (LHW) and non-catalyzed and catalyzed steam explosion (SE) are practical and versatile methods, since there is minimal need for catalysts or special reactor materials or preliminary reduction of the feedstock size [14]. 

## 2. Liquid Hot Water Pretreatment

Liquid hot water pretreatment (LHW) is one of the legacy technologies used for pretreatment of cellulosic raw materials. This process exposes a raw material to high-temperature cooking in water under high pressure. Pressure is used to keep water in a liquid state at elevated temperatures of 160–240 °C [14,15,16].

The purpose of the LHW pretreatment is to remove hemicelluloses, make cellulose more accessible for interaction with an enzyme, and prevent inhibitors from formation. During LHW, hemicelluloses are basically depolymerized, and their degradation products dissolve in the liquid fraction. Cellulose is completely retained in the solid fraction. Lignin concurrently undergoes depolymerization and repolymerization reactions [17]. It was found that lignin remains on the surface of the treated biomass after the LHW treatment due to re-condensation of soluble lignin components [18], which may have a negative impact on enzymatic hydrolysis because of enzyme adsorption [19].

The LHW pretreatment efficiency depends on the feedstock nature, including lignin content, cellulose fiber crystallinity degree, and complexity of the cellulose–hemicellulose matrix [20]. The suspension resulting from the LHW pretreatment can be filtered off to obtain two fractions: a cellulose-rich solid fraction and a liquid fraction enriched with sugars derived from hemicelluloses [21].

The critical factors in the LHW pretreatment are temperature and holding time [22,23,24,25,26]. From the example of methane production, the maximal yield of reducing sugars was achieved at 175 °C for 30 min at different temperatures (150 to 225 °C) and time (5 to 60 min), which is a 62.9% increase compared to untreated straw [23]. In a similar study [22], the highest hemicellulose removal and the maximal biomass hydrolysis after LHW were achieved at 180 °C and at a holding time of 30 min. The LHW pretreatment of willow sawdust at different temperatures (130 to 230 °C) showed that temperatures above 200 °C led to the high solubility of celluloses in the liquid fraction and to the formation of furfural, 5-hydroxymethylfurfural and organic acids as compared to lower treatment temperatures [25]. The highest yield of reducing was obtained by the LHW treatment of coffee cherry at a temperature of 180 °C for 5 h [26]. The LHW pretreatment of corn cobs at 160 °C for 10 min provided a maximum pentose yield of 58.8% in the liquid fraction, removal of lignin from the solid fraction by more than 60%, and a 73.1% yield of glucose during enzymatic hydrolysis [27]. A study on the production of L-lactic acid showed that the optimal efficiency of enzymatic hydrolysis when corn husk was pretreated with LHW was achieved at 155 °C for 15 min [28]. The LHW treatment of pineapple leaves at 160 °C for 60 min resulted in maximal yields of xylooligosaccharides and glucooligosaccharides of 23.7 and 18.3%, respectively, in the liquid fraction [29].

The LHW pretreatment of cellulosic raw materials at a controlled pH promotes efficient hemicellulose hydrolysis and lignin depolymerization, minimizing the formation of monosaccharides and their further degradation to toxic compounds [30,31]. To avoid the formation of toxic compounds during the pretreatment, the pH should be maintained between 4 and 7, because this pH preserves hemicellulosic sugars in oligomeric form and minimizes the formation of monomers [30]. The other studies [30,31,32] reported that the pH of LHW-pretreated cellulosic raw materials is usually within the range of 4–5 without adding a base or buffer [30,31,32].

The study [33] proposed using a ball mill for grinding and further treating corn straw with LHW. However, the results showed that there is no correlation between grinding and enzymatic hydrolyzability of treated biomass. The solid loading also has an impact on the LHW treatment efficiency. In the study [34], the highest ethanol yield of 172.1 L/ton was obtained at a solid loading of 5%.

The severity factor during the LHW process also affects the chemical composition of the treated feedstock and its ability to undergo enzymatic hydrolysis [17]. Low temperature and long duration are preferable for the removal of hemicelluloses during pretreatment because these conditions favor the formation of oligosaccharides and monomeric sugars rather than sugar degradation to toxic compounds [35]. In contrast, high cellulose hydrolyzability is achieved at a high temperature (>200 °C) and a high pretreatment severity when pretreatment is followed by enzymatic hydrolysis [36]. Thus, the LHW pretreatment conditions depend on a specific raw material (Table 1).

The two-stage LHW pretreatment was developed to fully extract hemicelluloses into the liquid fraction and enhance the reactivity of the solid fraction to enzymatic hydrolysis. The maximal total yield of xylose was 86.4%, and the recovery of reducing sugars after 72 h enzymatic hydrolysis reached 96.63%, which is superior to the recovery by the one-step pretreatment with hot water or dilute acid [48]. It was also found that the hot or cold washing of the solid fraction following the LHW pretreatment of biomass increases the yield of reducing sugars by 10–35%. The reasons behind this phenomenon have not been established, although there is a possibility that dissolved phenolic compounds, acetic acid and sugars resulting from the hemicellulose hydrolysis might have an inhibitory effect on cellulases contained in enzyme cocktails, which may lead to a low yield of reducing sugars [32]. Despite the presence of toxic compounds, the LHW pretreatment method is not inferior to the other pretreatments with respect to the yield of reducing sugars. The 72 h enzymatic hydrolysis of pretreated sugarcane bagasse reached 71.6% for the LHW process, 76.6% for hydrochloric acid pretreatment, and 77.3% for sodium hydroxide pretreatment [49]. That said, the resultant hydrolyzates are considered biologically good quality and suitable for further production of high-value-added products [22,28,34,37,40,43,44,46].

The LHW treatment is used in combination with other pretreatment methods; for example, LHW pretreatment combined with aqueous ammonia [50], carbon dioxide [51], sodium carbonate and oxygen [52,53], biological pretreatment [54], organosolv pretreatment [35], lactic acid [55,56], and others [57,58]. The combined pretreatment methods enhance the enzymatic hydrolyzability of treated biomass. 

The LHW pretreatment does not require addition of chemicals and catalysts; therefore, an inexpensive reactor design can be used because of the low corrosion potential. There is no neutralization stage for the spent liquor. There is no need to incorporate the grinding stage of cellulosic feedstocks, which is a high-cost operation, especially for industrial scale applications, since the LHW process creates an effective separation between the particles. In addition, the great advantage is that a high pentose recovery is achieved, with fewer inhibitors being formed. The main disadvantages of the LHW process are high energy inputs due to the high pressure and large amount of water required by the system. Also, the disadvantage is that lignin is negligibly removed from the solid fraction. Overall, the LHW pretreatment has high economic potential and is simple and understandable, which is an advantage for the integration and development of the process. Thus, this pretreatment method has sufficient potential for commercialization.

## 3. Non-Catalyzed Steam Explosion Pretreatment

Steam explosion (SE) is the most widely used physicochemical method for pretreatment of cellulosic raw materials [59,60]. In the non-catalyzed SE process, a feedstock is pretreated with steam at moderate temperature and pressure for a certain time. The pressure is then quickly released to expand the biomass fibers [21,61]. The steam penetrates through the plant cell wall, causing destruction of the multi-level structure of cellulosic raw materials. It was noted that organic acids like acetic and formic acids are formed during steam explosion [62]. Acetic acid liberated from the acetylated hemicellulose and water both catalyze the hydrolysis of hemicellulose to oligosaccharides, which are then partially hydrolyzed to monosaccharides and remain in the liquid fraction [63,64]. Cellulose undergoes little change during the SE pretreatment, due to its high crystallinity degree and the presence of inaccessible microfibrils [14], and lignin is degraded by the high temperature [65].

In this way, SE promotes a reduction in the cellulose crystallinity, an increase in the porosity [66,67], a reduction in the biomass particle size [64], and delignification [16]. Studies [65,68,69] have shown that SE increases the reactivity to enzymatic hydrolysis several times compared to the untreated feedstock. However, the researchers observed the condensation of lignin during SE to form a more resistant compound [70]. The cellulose degradation during SE can be minimized by removing the biomass from the liquid fraction [71] or by applying the two-stage steam pretreatment [72].

The hydrolysis process of hemicelluloses generates aromatic compounds [73]. Lignin-related compounds also transform into chemical substances that can inhibit the subsequent processes. These inhibitors can be schematically classified into organic acids, furans, and phenolic compounds, according to their chemical structure. The fermentation inhibitors include furan derivatives like furfural and 5-hydroxymethyl furfural (5-HMF); aliphatic acids like acetic acid, formic acid, and levulinic acid; and phenolic compounds. Furfural and 5-hydroxymethyl furfural (5-HMF) are decomposition products from pentoses and hexoses. Furfural is produced by the Maillard reaction and occurs as a byproduct during the high-temperature hydrolysis of cellulosic matter. Furfural and 5-HMF can be further cleaved to formic and levulinic acids [74]. There is data indicating that these products are strong inhibitors of microbial growth (the chemical nature of phenolic compounds determines their toxicity and induces different physiological responses in *Saccharomyces cerevisiae* in hydrolyzates) [75] and that detoxification strategies need to be devised in order to enhance the fermentability of hydrolyzates into various biotechnology products [2]. To address this problem, scientists are developing strategies to counteract lignocellulose-derived inhibitors that are released during pretreatment: biomass selection and modification (selection of a suitable feedstock and/or technologies that produce fewer undesirable compounds); detoxification/conditioning (chemical additives, e.g., alkaline, BSA, polymers, activated carbon); biological detoxification (use of microbes); microbial adaptation (adaptive evolution of a particular microbe in an inhibitory environment); and genetic/metabolic engineering (use of genetically modified microbes for lignocellulosic hydrolyzates) [2]. The main methods of enhancing the adaptive potential of microorganisms may include maintenance of pH homeostasis and cell membrane integrity, the activation of global stress responses, and inhibitor degradation [76]. In [77], after sugarcane bagasse was pretreated by SE, the alkaline delignification stage was carried out to reduce the inhibitory effect of lignin on enzyme cocktails. The obtained results suggest that the mass content of cellulose increased to about 87% on average, and the removal of hemicellulose and lignin exceeded 90% in that study.

The SE pretreatment efficiency depends on factors like temperature, pretreatment time, feedstock particle size, and moisture content [70]. In [78], SE at 200 °C for 10 min led to a high yield of reducing sugars by enzymatic hydrolysis (91.7%) and to a total glucose yield of 35.4 g glucose/100 g wheat straw, with a high amount of toxic compounds being formed. Temperatures below 200 °C contributed to the minimal degradation of sugars and formation of toxic compounds during SE. Similar results were obtained in the study [79]: maximal yields of glucose in enzymatic hydrolysis were obtained after pretreatment of raw materials at 210 °C for 10 min. As to the xylose yield in the liquid fraction, it increased at temperatures up to 190 °C and at a longer process time. Temperatures higher than 190 °C favored the formation of furfural. It was found that, on average, about 3.71 wt.% of cellulose and 46.62 wt.% of hemicellulose turned into glucose and xylose, respectively, and about 26.73 wt.% of lignin turned into other chemicals at a temperature of 200 °C for 5 min [80]. It is worth noting that SE processes performed in the temperature range from 200 to 280 °C and at a holding time from 2 to 10 min promote an increase in the hemicellulose proportion and lignin removal, but the thermal decomposition of cellulose to sugars intensifies in this case [81]. In [82], the best yield of glucose (86%) during enzymatic hydrolysis was achieved at a temperature of 210 °C and a holding time of 15 min, equivalent to 19.76 g of glucose per 100 g of dry raw material. However, the optimized temperature-to-time ratio (270 °C for 1 min or 190 °C for 10 min) may have an effect on the SE efficiency [83].

Apart from the temperature and holding time, the particle size and moisture content of cellulosic feedstocks also influence the SE efficiency [84,85]. One study [86] showed that small particles (0.5, 0.25 and smaller than 0.25 mm) had lower lignin content after SE and higher enzymatic hydrolysis ability than larger particles (1–4 mm). The removal of hemicelluloses and conversion of the resultant biomass into reducing sugars after SE with particle sizes of 2.5, 2.0, 2.0, 1.5, 1.0 and 0.5 cm were compared. The highest removal of hemicelluloses from the feedstock was achieved at particle sizes of 1.0 and 0.5 cm, respectively, but the highest conversion of biomass into reducing sugars was achieved at a particle size of 2.5 cm. In this way, using the larger feedstock particles allowed the reduction of the process cost due to better mixing, as compared to the small particles [87]. Steam treatment of standard-sized aspen chips (*Populus tremuloides*) at 180 °C for 4 to 18 min showed that the uniform treatment of large-sized wood chips is possible [88]. Studies showed that the SE treatment of sugar cane bagasse samples with high moisture content provided a significant increase in the recovery of cellulose, hemicelluloses and lignin compared to samples with low moisture content. On the other hand, the increase in moisture content had a negative effect on the yield of reducing sugars during enzymatic hydrolysis of cellulose [89]. Similar conclusions were reached in [90]. Besides the above-listed factors, the SE efficiency is influenced by the explosion power density (EPD) [91]. This parameter is the subject matter of separate studies. Thus, the SE pretreatment conditions, as in the case of LHW, depend on a specific feedstock (Table 2).

The non-catalyzed SE pretreatment is considered an environmentally friendly and efficient method, as it does not use chemicals and requires less energy input for processing. The cost of the whole process is significantly reduced because different particle sizes can be used. However, it has been found that this method is applicable to cellulosic raw materials with low lignin content. Cellulosic raw materials containing large amounts of lignin are less amenable to this pretreatment. The drawbacks also include incomplete cleavage of lignin–hemicellulose linkages, leading to the release of complexes that may condense and precipitate to reduce the total recovery of sugar. In fact, SE is observed to partially degrade hemicellulose and generate some toxic compounds, such as furfural, hydroxymethyl furfural (due to degradation of sugars) and levulinic acid, vanillic acid, caproic acid, caprylic acid, pelargonic acid and palmitic acid (due to lignin degradation), which may affect enzymatic hydrolysis and fermentation.

## 4. Catalyzed Steam Explosion Pretreatment

In some instances, some gaseous/liquid chemicals like sulfur dioxide, sulfuric acid, ammonia and carbon dioxide are added as catalysts to impregnate the feedstock prior to SE [105]. The addition of a catalyst during SE helps not only to reduce the process duration and temperature, but also to improve the subsequent enzymatic hydrolysis of the treated biomass, lower the formation of inhibitory compounds and enhance hemicellulose removal [106]. The use of sulfur dioxide in SE is regarded as one of the most cost-effective pretreatment processes. Sulfur dioxide is employed to pre-soak the raw material prior to the SE pretreatment. The SE pretreatment after sulfur dioxide impregnation is an effective method to hydrolyze hemicelluloses and soften the cellulose structure, in order to enhance the reactivity to enzymatic hydrolysis. In [106], the use of sulfur dioxide as a catalyst during SE increased the reactivity of biomass to enzymatic hydrolysis compared to SE without a catalyst (60.3–68.3% vs. 24.2–43.9%, respectively). However, the use of sulfur dioxide generated a high content of inhibitory compounds. It was discovered that increasing the SE temperature, duration and sulfur dioxide content provided a decrease in the cellulose mass content from 47.62% to 18.64% [107].

The use of sulfuric acid as a catalyst in SE of wood showed that the best xylose yield in the liquid fraction (almost 70%) was obtained after pretreatment with 1.5% sulfuric acid at 200 °C for 5 min. Under harsher conditions, the xylose yield declined [108]. The study on the effect of the sulfuric acid concentration on SE showed that the cellulose yield decreased as the concentration was raised. The optimal concentration of sulfuric acid was 0.25% (vol./vol.). Presumably, increasing the acid concentration caused cellulose degradation [109]. In another study [110], the optimal concentration of sulfuric acid was 1.45%, and the cellulose-to-glucose conversion attained 78%. 

Besides sulfuric acid, phosphoric acid can also be used in SE. The comparative pretreatment of sugar cane bagasse with sulfuric and phosphoric acids showed that phosphoric acid is more preferable, because the obtained substrates are more susceptible to enzymatic hydrolysis [111].

The benefits of the sulfur dioxide and sulfuric acid treatments are the possibility to reduce the reaction time and temperature, increase the enzymatic hydrolysis rate, reduce the inhibitor formation, and completely remove hemicellulose and lignin. However, sulfur dioxide is more preferable, due to its low corrosiveness and low cost. Sulfuric acid is toxic and increases occupational risks.

Ammonia fiber explosion (AFEX) is an alkaline pretreatment process that causes physicochemical changes in the structure of cellulosic feedstocks [112]. In the AFEX process, the biomass is exposed to liquid anhydrous ammonia under high pressure and temperature (60–100 °C) for 5 to 30 min, followed by pressure release. The AFEX pretreatment decrystallizes cellulose, partially depolymerizes hemicellulose, removes acetyl groups chiefly on hemicellulose, cleaves lignin–carbohydrate complex linkages, cleaves the lignin C-O-C bond, and increases the accessible surface area, due to structural disruption and enhanced wettability of the treated biomass [112]. It was discovered that the AFEX pretreatment of sugarcane bagasse and cane leaf residues provides up to 85% conversion of cellulose and up to 95–98% conversion of xylan into sugars [113].

During the AFEX process, decomposition products like furans, phenols and other organic acids are formed. These products can inhibit microbial synthesis. However, compared to the dilute H_2_SO_4_ pretreatment, AFEX produces fewer inhibitory compounds [114].

The influencing factors of the AFEX process are treatment time, temperature, ammonia-to-biomass ratio, and moisture content. These parameters vary, depending on the type, growth location and harvesting time of biomass [115]. For example, for *Miscanthus giganteus*, the optimal AFEX parameters to maximize the cellulose conversion are as follows: 160 °C, 2:1 (g/g) ammonia-to-biomass ratio, 233% moisture content (dry weight), and 5 min reaction time for water-impregnated *Miscanthus* [116]. When sugarcane baggase was processed by the AFEX method, the maximal conversion of glucan and xylan was achieved at 100 and 120 °C [113], while in switch-grass processing, the optimal temperature was 80 °C, at which the maximal achieved sugar yield was 62.5% [115]. It is also important to note that the AFEX process efficiency is influenced by particle size [117]. The study on the effect of particle size (2 mm, 5 mm, vs. 2 cm and 5 cm) on the rice straw conversion demonstrated that larger rice straw particles (5 cm) exhibit a higher cellulose conversion compared to small particles during enzymatic hydrolysis [118]. For each type of feedstock, the above-mentioned parameters need to be optimized [119].

The advantage of the AFEX pretreatment is the high yield of reducing sugars (up to 80–90%) and the low formation of inhibitory compounds (compared to the other pretreatment methods). The ammonia used in this process can be recovered and reused. The disadvantage is the high cost of equipment, including pressure and temperature sensors, and transducers. The equipment must be made of materials that do not react with ammonia to prevent corrosion in the presence of highly alkaline mixtures of ammonia and water. Also, the use of large volumes of ammonia and the need to recycle ammonia to avoid environment harm incur extra costs.

Supercritical carbon dioxide (SC-CO_2_) explosion is a pretreatment method that uses a supercritical fluid as a “green solvent”, which represents a material that exists at a temperature and pressure above its critical point, where the gaseous and liquid phases are indistinguishable [120]. Carbon dioxide molecules are able to penetrate the fine pores of cellulosic raw materials, just like water and ammonia in the AFEX and SE pretreatments [121,122].

The carbon dioxide explosion alters the biomass structure, reduces the crystallinity degree and enhances the cellulose permeability, accessibility and surface area, thereby increasing the yield of reducing sugars after enzymatic hydrolysis, as opposed to untreated biomass [123]. The SC-CO_2_ pretreatment of corn stover at 150 °C and 24.1 MPa pressure for 1 h at a moisture content of 75% allows for a glucose yield of 30%, which is 18% higher than that of untreated corn [124].

The parameters influencing the process are temperature, pressure, process time, moisture content, and ratio of SC-CO_2_ to biomass. The results demonstrate that the optimal conditions for the SC-CO_2_ treatment of sugar cane bagasse to obtain 360 g/kg of fermentable sugars are as follows: 80 °C temperature, 250 bar pressure, 65 wt.% moisture, and 120 min duration [125]. When rice straw was treated with SC-CO_2_, the glucose yield from enzymatic hydrolysis increased as the pressure was raised from 0 to 30 MPa, showing that the pressure rise positively influences the enzymatic hydrolysis process [123,126]. The same conclusions were reached in a similar study [127]. When the temperature was raised from 40 °C to 110 °C during the SC-CO_2_ treatment, the yield of reducing sugars rose from 29.0% to 32.4% [128]. However, the SC-CO_2_ treatment of sorghum at temperatures of 30 °C, 50 °C and 70 °C showed that the yield of reducing sugars increased up to 50 °C and then declined [129]. The SC-CO_2_ treatment of bagasse at a pressure of 14 MPa for 5 to 60 min led to an increase in the yield of reducing sugars, from 27.0% to 72.0% [130]. The studies performed at different moisture contents of pine treated with SC-CO_2_ demonstrated that the yield of reducing sugars rose from 15% to 79.0% as the moisture content was raised from 0% to 73.0% [131,132].

The advantages of the SC-CO_2_ pretreatment include high availability, low cost, low corrosiveness, low occupational risks, low inhibitor formation (compared to steam explosion), and no environmental impact (compared to AFEX), since CO_2_ can be reused through photosynthesis. The disadvantages are high maintenance costs to create higher pressures (24 MPa if necessary) and the expensive reactor in order to operate with CO_2_ under pressure.

**Figure 1:**
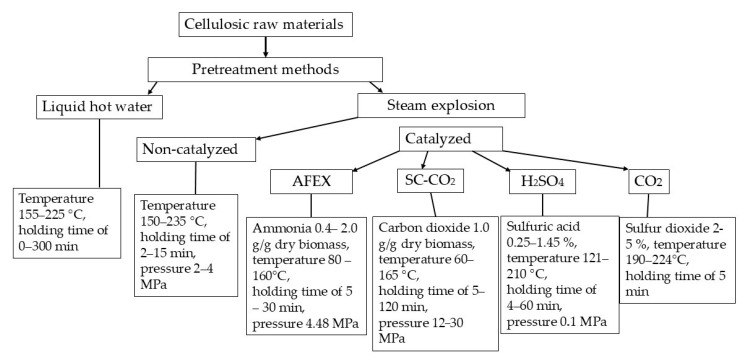
Features of the LHW and non-catalyzed and catalyzed SE methods.

Next, Figure 1 displays the features of the LHW and non-catalyzed and catalyzed SE methods, and Table 3 shows advantages and disadvantages of these methods.

## 5. Techno-Economic Analysis or Life Cycle Assessment of Liquid Hot Water and Steam Explosion Methods

It was established in the life cycle assessment of ethanol produced in a biorefinery from LHW-pretreated switch-grass that the biorefinery strategy for producing ethanol, electricity, furfural, acetic acid, and formic acid showed better results in terms of environmental impact, and turned out to be the best process, from a technical and economic perspective, when compared to the production of only ethanol and electricity [133,134].

The techno-economic analysis of the SE and AFEX processes demonstrated that at the current level of technology, these methods are cost-competitive, due to their close production costs (USD/kg) of 0.43 and 0.65 for sugar, respectively [80].

The life cycle assessment analysis of bioethanol production from corn straw showed that SE is one of the most environmentally friendly methods, with an especially low potential for eutrophication and acidification due to the short residence time of only 2 min. The exception to this was CO_2_ emissions, where they were ten times higher than the lowest simulated LHW emissions. LHW had very high sugar conversion rates, reaching 90.5% and 81.8% for glucan and xylan, respectively. The process was environmentally favorable compared to other pretreatments, because this method used only deionized water under pressure to pretreat the corn straw. This technique provided the lowest CO_2_ emissions. It can be concluded that LHW is the most suitable pretreatment method for corn straw. The application of this method will improve long-term energy security and create a greener future [135].

The life cycle assessment approach was applied to evaluate the environmental feasibility of the conversion of *Cynara cardunculus* into bioethanol using different types of biomass pretreatment. The SE pretreatment process outperforms the dilute acid pretreatment process among all analyzed treatments. The combination of SE and subsequent alkaline extraction can reduce energy inputs and therefore improve the environmental performance of the process in terms of the energy-related exposures [136].

## 6. Conclusions

To sum up, pretreatment is needed to break down the plant matrix of cellulosic raw materials and liberate fermentable sugars that can be further transformed into value-added products. Pretreatment can remove most of the hemicellulose and partially dissolve the lignin, increasing the accessibility of cellulose to enzyme and enhancing the yield of sugars. The present review described pretreatment methods such as liquid hot water (LHW) and steam explosion (SE). The features of these processes, as well as benefits and drawbacks of the methods, were also listed herein. The efficiency of these pretreatments and their impact on cellulosic raw materials strongly depends on the type of feedstock (component composition), pretreatment method, and pretreatment conditions (pressure, temperature, duration, etc.). Thus, the efficient LHW and SE pretreatment processes should take account of the properties and characteristics of a specific cellulose-containing vegetative feedstock, in order to improve the cellulose yield. The life cycle assessment approach demonstrated that the SE pretreatment outperforms dilute acid pretreatment methods and lowers energy inputs, and hence improves the environmental performance of the process, while the LHW method improves long-term energy security and creates a greener future.

## Figures and Tables

**Table 1:** Cellulosic feedstocks treated by the LHW method.

Initial Feedstock	Pretreatment Conditions	Product	Ref.
Bamboo (*Neosino calamus affinis*)	solid-to-water ratio of 1:10 (wt./vol.), temperature of 170 °C	Bioethanol	[37]
*Caragana korshinskii*	solid-to-water ratio of 1:20 (wt./vol.), temperature of 160 °C, holding time of 120 min, stirring speed of 150 rpm	-	[38]
Coffee cherry	solid-to-water ratio of 1:9 (wt./vol.), temperature of 180 °C, holding time of 5 h	-	[26]
Corncobs	solid content of 10% (by weight), temperature of 160 °C, holding time of 10 min, initial pressure of 25 bar under nitrogen	-	[27]
	solid content of 10% (by weight), temperature of 200 °C, holding time of 30 min, stirring speed of 150 rpm	-	[39]
Corn husks	solid-to-water ratio of 1:20 (wt./vol.), temperature of 155 °C, holding time of 15 min	lactic acid	[28]
Corn Stover	solid-to-water ratio of 1:15 (wt./vol.), temperature of 162.4 °C, holding time of 29.5 min	bioethanol	[40]
Empty palm fruit bunch	solid content of 15% (by weight), temperature of 185 °C, holding time of 0 min	ethanol	[34]
	temperature of 121 °C, holding time of 60 min	ethanol	[41]
Hazelnut tree pruning residue	solid-to-water ratio of 1:10 (wt./vol.), temperature of 210 °C, holding time of 45 min, stirring speed of 300 rpm	-	[42]
Olive stones	temperature of 225 °C, holding time of 0 min	ethanol	[43]
Pineapple leaves	solid-to-water ratio of 1:10 (wt./vol.), temperature of 160 °C, holding time of 60 min	-	[29]
*Quercus mongolica*	solid-to-water ratio of 1:8 (wt./vol.), temperature of 200 °C, holding time of 10 min	succinic acid	[44]
*Sida hermaphrodita* (L.)	temperature of 180 °C, holding time of 30 min, stirring speed of 13,000 rpm	methane	[22]
Switchgrass	solid content of 15% (by weight), temperature of 200 °C, holding time of 5 min	-	[45]
Rice straw	solid content of 20.0% (by weight), temperature of 180 °C, holding time of 10 min, stirring speed of 500 rpm	methane	[46]
Waste wheat straw	solid-to-water ratio of 1:10–1:500 (wt./vol.), temperature of 180 °C, holding time of 10 min	-	[47]

**Table 2:** Cellulosic feedstocks treated by the SE methods.

Initial Feedstock	Pretreatment Conditions	Product	Ref.
Bamboo	solid-to-water ratio of 1:10 (wt./vol.), temperature of 230 °C, holding time of 3 min	-	[92]
Common reed (*Phragmites australis*)	pressure of 3.4 MPa, temperature of 200 °C; holding time of 15 min	biogas	[93]
Corn stalk	temperature of 190 °C, pressure of 1.37 MPa, holding time of 5–10 min	-	[91]
*Eucalyptus globulus*	temperature of 198 °C, temperature of 5 min	-	[94]
Hay	temperature of 220 °C, holding time of 15 min	biogas	[95]
Giant reed (*Arundo donax* L.)	pressure of 4 MPa, temperature of 204 °C, holding time of 9.5 min	-	[96]
Microalgae	temperature of 150 °C, holding time of 5 min	bioethanol	[97]
*Miscanthus* × *giganteus*	-	bioethanol	[98]
Olive tree pruning	pressure of 2 MPa, temperature of 210 °C, holding time of 15 min, severity factor of 4.41	-	[82]
Palm empty fruit bunches	temperature of 195 °C, holding time of 6 min	-	[99]
	pressure of 1.5 MPa, holding time of 1 min		[100]
Poplar	-	bioethanol	[98]
Rapeseed straw	temperature of 215 °C, holding time of 7.5 min	bioethanol	[101]
Rice straw	temperature of 220 °C, holding time of 2 min	-	[102]
Sisal	pressure of 137 Pa, holding time of 60 min	cellulose nano fibers	[103]
Spruce wood chips	temperature of 235 °C, pressure of 3.1 MPa, holding time of 10 min	-	[104]
Wheat straw	-	bioethanol	[98]
	solid-to-water ratio of 1:10 (wt./vol.), 60% moisture of starting material, temperature of 200 °C, holding time of 10 min	sugars	[78]

**Table 3:** Advantages and disadvantages of the LHW and non-catalyzed and catalyzed SE methods.

	Pretreatment Methods					
	Liquid Hot Water	Steam Explosion				
		Non-Catalyzed	Catalyzed			
			SO_2_	H_2_SO_4_	AFEX	SC-CO_2_
Grinding	−	−	−	−	−	−
Addition of chemicals and catalysts	−	−	+	+	+	+
Utilization of low-cost reactor design	−	−	+	+	+	+
Hemicelluloses passing into the liquid fraction	+	+	+	+	+	+
Removal of lignin from the solid fraction	insignificant	insignificant	complete	complete	complete	complete
Formation of inhibitors	insignificant	+	+	+	+	insignificant
High energy inputs and costs for equipment	+	+	−	−	+	+
Harmful environmental impact	−	−	+	+	+	−

− missing, + present.

## Data Availability

Data are contained within the article.
